# 2774. Predictors of Treatment Outcomes in Bloodstream Infections Due to OXA-48-producing *Klebsiella pneumoniae*

**DOI:** 10.1093/ofid/ofad500.2385

**Published:** 2023-11-27

**Authors:** Po-Han Huang, Sheng-Hua Chou, Wen-Yin Chen, Yi-Tsung Lin

**Affiliations:** Division of Infectious Diseases, Department of Medicine, Taipei Veterans General Hospital, Pittsburgh, Pennsylvania; Institute of Emergency and Critical Care Medicine, National Yang-Ming University, Taipei, Taiwan, Taipei, Taipei, Taiwan (Republic of China); Taipei Heart Clinic, Taipei, Taipei, Taiwan; Division of Infectious Diseases, Department of Medicine, Taipei Veterans General Hospital, Taipei, Taiwan, Taipei, Taipei, Taiwan (Republic of China)

## Abstract

**Background:**

*Klebsiella pneumoniae* causes various infections and the rising prevalence of carbapenem-resistant *K. pneumoniae* (CRKP) constitutes a major public health threat. Carbapenemase production is the most common mechanism of carbapenem resistance in CRKP, and OXA-48-like-producing strains accounted for about one-fifth of carbapenemase-producing CRKP worldwide. Despite clinical evidence supporting the use of ceftazidime-avibactam (CZA) in treating carbapenem-resistant *Enterobacterales* (CRE) infections, studies regarding its effectiveness for OXA-48-producing CRKP is limited. OXA-48-producing CRKP may test susceptible to meropenem in vitro, but whether meropenem is effective when phenotypic-genotypic discordance exists is unknown.

**Methods:**

In this retrospective cohort study, we evaluated patients with OXA-48-producing CRKP bacteremia at Taipei Veterans General Hospital from April 2017 through December 2022. Patients were categorized into five groups according to the treatment received: CZA, meropenem, colistin, colistin-meropenem combination, and other treatments. Clinical outcomes were compared according to the antibiotics received, and logistic regression was performed to evaluate risk factors associated with treatment failure.

**Results:**

Forty-five patients were included, with 87% (39/45) having received at least one active antibiotic against the OXA-48-producing CRKP isolated from blood. Fourteen- and 30-day mortality rates were 31% and 38%, respectively. The clinical failure rate was 27% (4/15) for patients treated with CZA, 40% (4/10) with meropenem, 100% (8/8) with colistin, 75% (3/4) with meropenem-colistin-combination, and 38% (3/8) with other treatments (P = 0.010). The clinical failure rate for meropenem-treated patients dropped to 25% (2/8) if only CRKP strains with meropenem minimal inhibitory concentration (MIC) ≤ 4 mg/L were analyzed. In multivariate analysis, clinical failure was associated with Apache II Score (P = 0.022) and colistin use (P = 0.002).

Rates of clinical failure across treatment regimens

**Figure:**
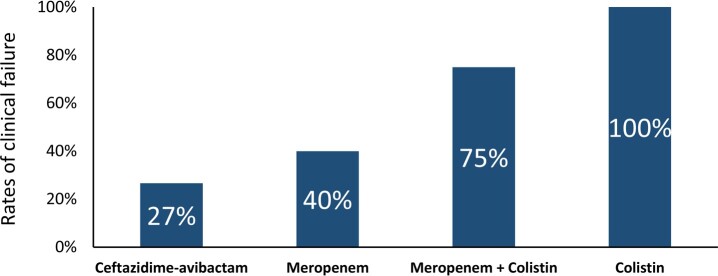

Rates of clinical failure at different meropenem MIC values in patients receiving meropenem treatment

**Figure:**
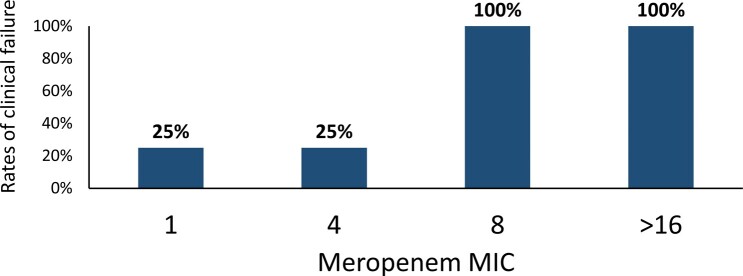

MIC, minimum inhibitory concentration

Multivariable analysis of risk factors associated with clinical failure at 30 days

**Figure:**
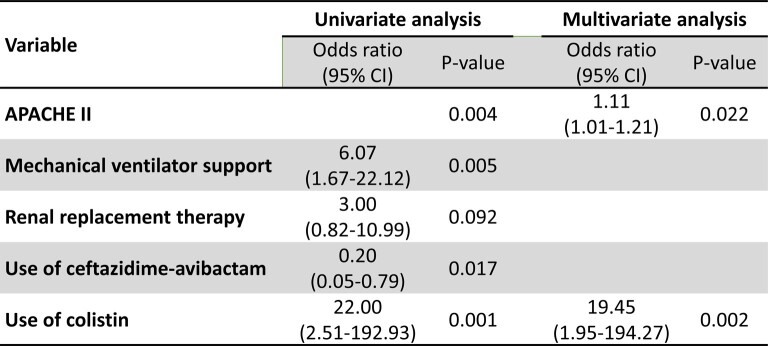

CI, conﬁdence interval

**Conclusion:**

CZA is an important option in the treatment of OXA-48-producing CRKP bacteremia, and meropenem may remain a reasonable alternative if meropenem MIC was ≤ 4 mg/L. Use of colistin is an independent risk factor for treatment failure and should be avoided.

**Disclosures:**

**Po-Han Huang, MD, MSc**, Pfizer: Honoraria

